# Identification of oxytocin-related lncRNAs and assessment of their expression in breast cancer

**DOI:** 10.1038/s41598-021-86097-2

**Published:** 2021-03-19

**Authors:** Sepehr Behtaji, Soudeh Ghafouri-Fard, Arezou Sayad, Ali Sattari, Mathieu Rederstorff, Mohammad Taheri

**Affiliations:** 1 Université de Lorraine, 54000 Nancy, France; 2 Department of Medical Genetics, School of Medicine, Shahid Beheshti University of Medical Sciences, Tehran, Iran; 3 Université de Lorraine, CNRS, IMoPA, 54000 Nancy, France; 4 Urology and Nephrology Research Center, Shahid Beheshti University of Medical Sciences, Tehran, Iran

**Keywords:** Cancer, Genetics, Biomarkers

## Abstract

Oxytocin is a neuropeptide released by the central nervous system. A number of studies have demonstrated the role of this neuropeptide in the pathogenesis of breast cancer. In the present project, we have identified mRNA coding genes and long non-coding RNAs (lncRNAs) that are associated with this pathway through an in-silico strategy, and measured their expression in a cohort of Iranian females affected with this type of malignancy. Expression levels of *OXTR*, *FOS*, *ITPR1*, *RCAN1*, *CAMK2D*, *CACNA2D* and *lnc_ZFP161* were significantly down-regulated in breast cancer tissues compared with nearby non-cancerous tissues. On the other hand, expression of *lnc_MTX2* was higher in breast cancer tissues compared with controls. Expression of *lnc_TNS1* and *lnc_FOXF1* were not different between these two kinds of samples. Expression of *CACNA2D* was associated with mitotic rate and PR status (P values = 3.02E−02 and 2.53E−02, respectively). Expression of other oxytocin-related genes was not associated with clinicopathological parameters. *FOS* and *ITPR1* had the highest AUC value among the oxytocin-related genes. Combination of expression profiles of all oxytocin-related genes increased the AUC value to 0.75. However, the combinatorial sensitivity and specificity values were lower than some individual genes. In the breast cancer tissues, the most robust correlations have been detected between *lnc_ZFP161*/ *lnc_FOXF1*, *CAMK2D*/ *lnc_ZFP161* and *CAMK2D* / *lnc_FOXF1* (r = 0.86, 0.71 and 0.64 respectively). In the non-cancerous tissues, the strongest correlation was detected between *lnc_FOXF1/lnc_MTX2 and lnc_ZFP161*/*CAMK2D* respectively (r = 0.78 and 0.65). Taken together, oxytocin-associated genes have been dysregulated in breast cancer tissues. Moreover, the correlation ratio between these genes is connected with the existence of cancer.

## Introduction

Oxytocin is a neuropeptide secreted from the central nervous system and has similar functions with the antidiuretic hormone vasopressin^1^. In addition to its functions in the physiology of uterus and milk secretion, oxytocin has been shown to affect carcinogenesis^1^. A former in vitro study has demonstrated the mitogenic effects of oxytocin on MCF7 cells indicating the possible role of this neuropeptide in the growth of breast cancer cells^2^. Yet, another study in MCF7 and T47D breast cancer cells has shown the inhibitory effect of oxytocin on estrogen-associated cell growth. This neuropeptide has also been shown to promote the suppressive impact of tamoxifen on cell proliferation. Moreover, expression of oxytocin receptor has been detected in these cell lines and MDA-MB-231 cells^3^. Subsequent investigations have verified anti-proliferative effects of oxytocin and have demonstrated the role of cyclic adenosine monophosphate protein kinase A in the mediation of these effects^4^. Further experiments in animal models of breast cancer have also verified such effects^5^. As a G protein-coupled receptor, oxytocin receptor exemplifies a fascinating target for cancer treatment since it partakes in the development of in breast cancer and is expressed by numerous breast cancer cell lines^6^. Yet, the underlying mechanisms of involvement of oxytocin receptor and its related pathways are not completely understood. In the present project, we have identified mRNA coding genes and long non-coding RNAs (lncRNAs) which are associated with this pathway through an in-silico strategy, then measured their expression in a cohort of Iranian females affected with this type of malignancy^7^. We hypothesized that oxytocin-related lncRNAs are involved in the pathogenesis of different histopathological types of breast cancer.

## Materials and methods

### Bioinformatics methods

GSE54002 dataset was downloaded from Gene Expression Omnibus database and preprocessed in R version 3.6.1 using limma package version 3.40.6. This dataset was selected as it contains expression data of an appropriate number of clinical samples prepared by laser capture microdissection (417 patients with breast cancer and 16 non-tumor tissues) (https://www.ncbi.nlm.nih.gov/geo/query/acc.cgi?acc=GSE54002). Gene expression matrix was obtained using the log2 values. Then, data was normalized using limma package. Differentially expressed genes (DEGs) between tumoral and normal tissues were assessed using Bayes methods and limma package. Raw P values were corrected using Benjamini and Hochberg methods. Cut-off criteria for identification of DEGs were P < 0.05 and logFC > 2 for up-regulated genes and logFC < -2 for down-regulated genes. Pathway Enrichment Analyses of DEGs were performed using https://amp.pharm.mssm.edu/Enrichr and KEGG database. PPI network was depicted and hub genes were recognized using STRING (https://string-db.org) and Cytoscape v3.8.1. Then, from the down-regulated genes, those being associated with oxytocin pathway were selected. Finally, lncRNAs associated with these genes were chosen based on the results of Khalil et al. study (GSE16226)^8^.

### Enrolled individuals

Expression of oxytocin-related genes were assessed in 69 pairs of breast cancer specimens and their matched nearby tissues. Samples were gathered from Farmanieh and Sina hospitals during 2017–2020, Tehran, Iran. The study protocol was approved by the ethical committee of Shahid Beheshti University of Medical Science and the study protocol was performed in accordance with the relevant guidelines (IR.SBMU.MSP.REC.1398.1010). Patients’ samples were excised before any chemotherapy or radiotherapy. Medical records were gathered to obtain histopathological and clinical data. Informed written consent forms were obtained from study participants.

### Expression assays

All tissue sections were subjected to RNA extraction using the RiboEx kit (GeneAll, Seoul, South Korea). Afterwards, 70–100 ng of RNA was used for production of cDNA using the ExcelRT Reverse Transcription Kit II (SMOBIO, Taiwan). Expressions of genes in breast cancer samples and nearby non-cancerous tissues were measured in the ABI step one plus PCR machine. Expression levels were normalized to transcripts of *GAPDH*. RealQ Plus 2 × PCR Master Mix (Ampliqon, Odense, Denmark) was used for making the reactions. Primers and amplicons characteristics are shown in Table 1.

**Table 1:** Primers and amplicons characteristics.

Name	Sequence	Primer length	PCR product (bp)
OXTR (F)	GGACGCCTTTCTTCTTCGTG	20	128 bp
OXTR (R)	CATGTAGATCCAGGGGTTGCAG	22	
CAMK2D (F)	AGAAGAGACTCGTGTGTGGC	20	100 bp
CAMK2D (R)	AATACAGGGTGGCTTGATGGG	21	
ITPR1 (F)	GACGCAGTGCTACTCAACAAAC	22	126 bp
ITPR1 (R)	CAAATGCAGGAGCTGGATCAC	21	
RCAN1 (F)	AGACTGAGTTTCTGGGAAAGGA	22	101 bp
RCAN1 (R)	CAGAAACTGCTTGTCTGGATTTG	23	
CACNA2D1 (F)	ACCACGTTTTACACTGTGCCC	21	101 bp
CACNA2D1 (R)	GAGATTTGGGGTTCTTTGGCTGA	23	
FOS (F)	TACTACCACTCACCCGCAGA	20	105 bp
FOS (R)	CGTGGGAATGAAGTTGGCAC	20	
LINC01116 (TALNEC2 or lincMTX2) (F)	AACGCTTTTGAATATGGGGAC	21	67 bp
LINC01116 (TALNEC2 or lincMTX2) (R)	CAATCACAGAGCTCTCCTTGC	21	
DIRC3(lincTNS1) (F)	GGGAGTATGCCTCCAGACAG	20	70 bp
DIRC3(lincTNS1) (R)	GTCGATCAGCAAGCTCAGTG	20	
LINC00667 (lincZFP161) (F)	AATTGGAAGGAAACACAGCC	20	55 bp
LINC00667 (lincZFP161) (R)	GACTGCAGGCCACAGACAG	19	
LincFOXF1(FENDRR) (F)	TAAAATTGCAGATCCTCCCG	20	58 bp
LincFOXF1(FENDRR) (R)	AACGTTCGCATTGGTTTAGC	20	
GAPDH (F)	CATCAAGAAGGTGGTGAAGCAG	22	120 bp
GAPDH (R)	GCGTCAAAGGTGGAGGAGTG	20	

### Statistical analyses

Statistical analyses were executed in the R environment. Transcript quantities of oxytocin-related genes were measured in relation to the *HPRT1* reference gene using the equation: $\frac{am{p}_{gene}^{-C{T}_{gene}}}{am{p}_{House keeping}^{-C{T}_{House keeping}}}$ . Afterwards, the acquired values were log2 transformed and utilized for subsequent analysis.

A comparison was made between non-cancerous and tumor tissues of patients, and the significance of the difference between mean values was appraised using the paired t-test. Correlations between expression levels of oxytocin-related genes were appraised through the calculation of Spearman correlation coefficients. In order to appraise of the diagnostic power of genes, receiver operating characteristic (ROC) curves were depicted. ROC curves were depicted using the methods described previously^9, 10^. For this purpose, Bayesian Generalized Linear Model (BayesGLM), Generalized Linear Model (GLM), and Linear Discriminant Analysis (LDA) were used to compute the sensitivity and specificity of each model. GLM is a generalization of linear regression with no constraint on the distribution models of response variables. BayesGLM is an approach to GLM using Bayesian inference, and LDA aims to find a linear combination of features that separates two or more classes of objects or events. Log 2 values of transcript quantities of all genes were used as the predictive features to train three machine learning models with tenfold cross validation to avoid overfitting. Area under curve (AUC) metric was computed to pick the best model. Finally, BayesGLM model was selected based on the previous test, and the model was trained for each gene separately to test the distinguishing power of specific genes. Chi-square test was used to assess the association between demographic/clinical data and transcript levels of oxytocin-associated genes. Genes with log2FC ≥ 1 (tumor tissues vs. non-cancerous tissues) were considered as up-regulated and those with log2FC ≤ − 1 were considered as down-regulated. The level of significance was set at P value < 0.05.

## Results

### Bioinformatics step

**Figure 1:**
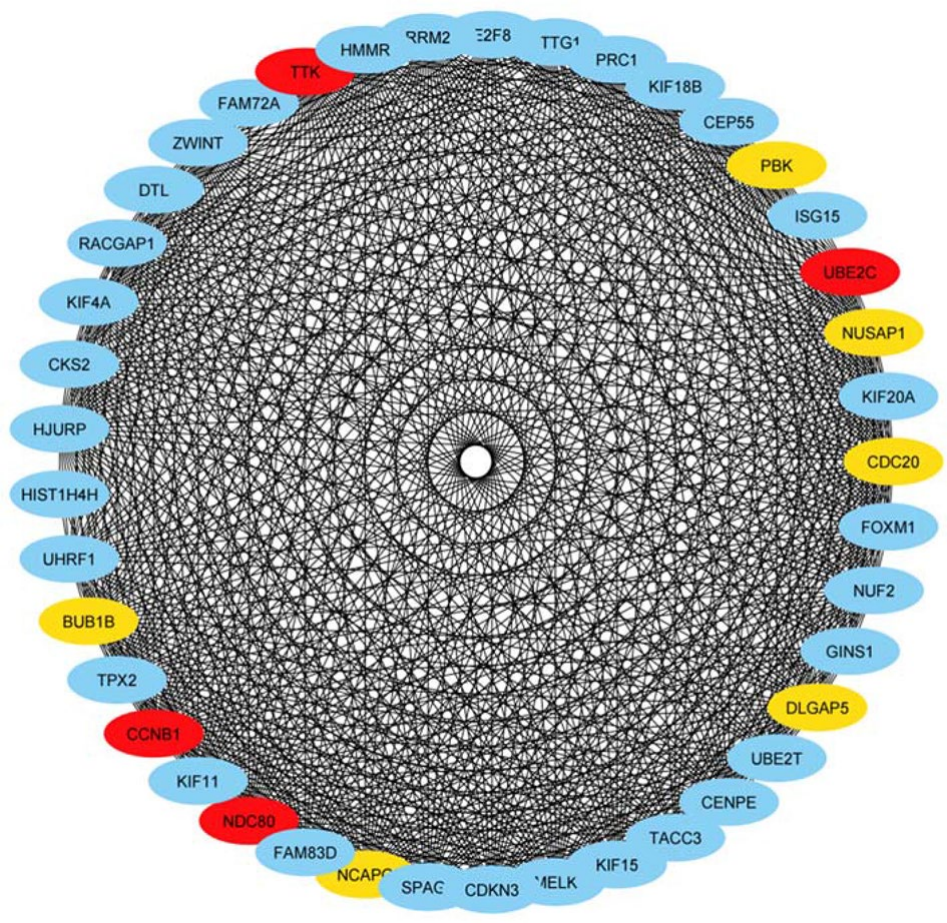
The interaction networks for down-regulated genes as predicted by STRING and visualized in Cytoscape. Hub genes and their neighbors are shown.

The in-silico method has led to identification of a number of down-regulated genes in cancerous tissues compared with non-cancerous tissues (Fig. 1).

**Figure 2:**
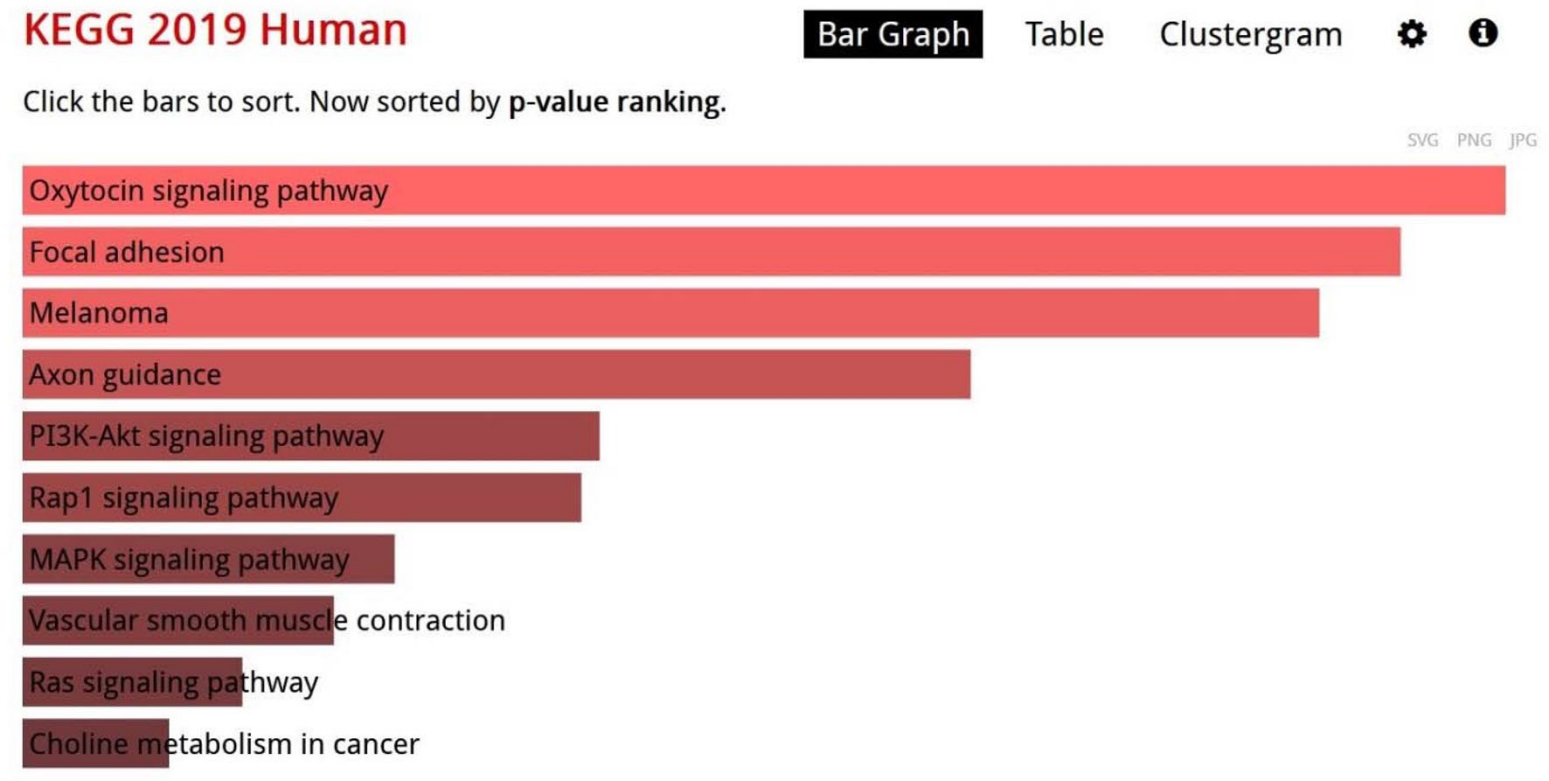
KEGG pathways of the enriched pathways of the down-regulated genes^11^.

KEGG pathways analysis revealed oxytocin signaling pathway as the most significant enriched pathway of the down-regulated genes (Fig. 2).

### Expression assays

**Figure 3:**
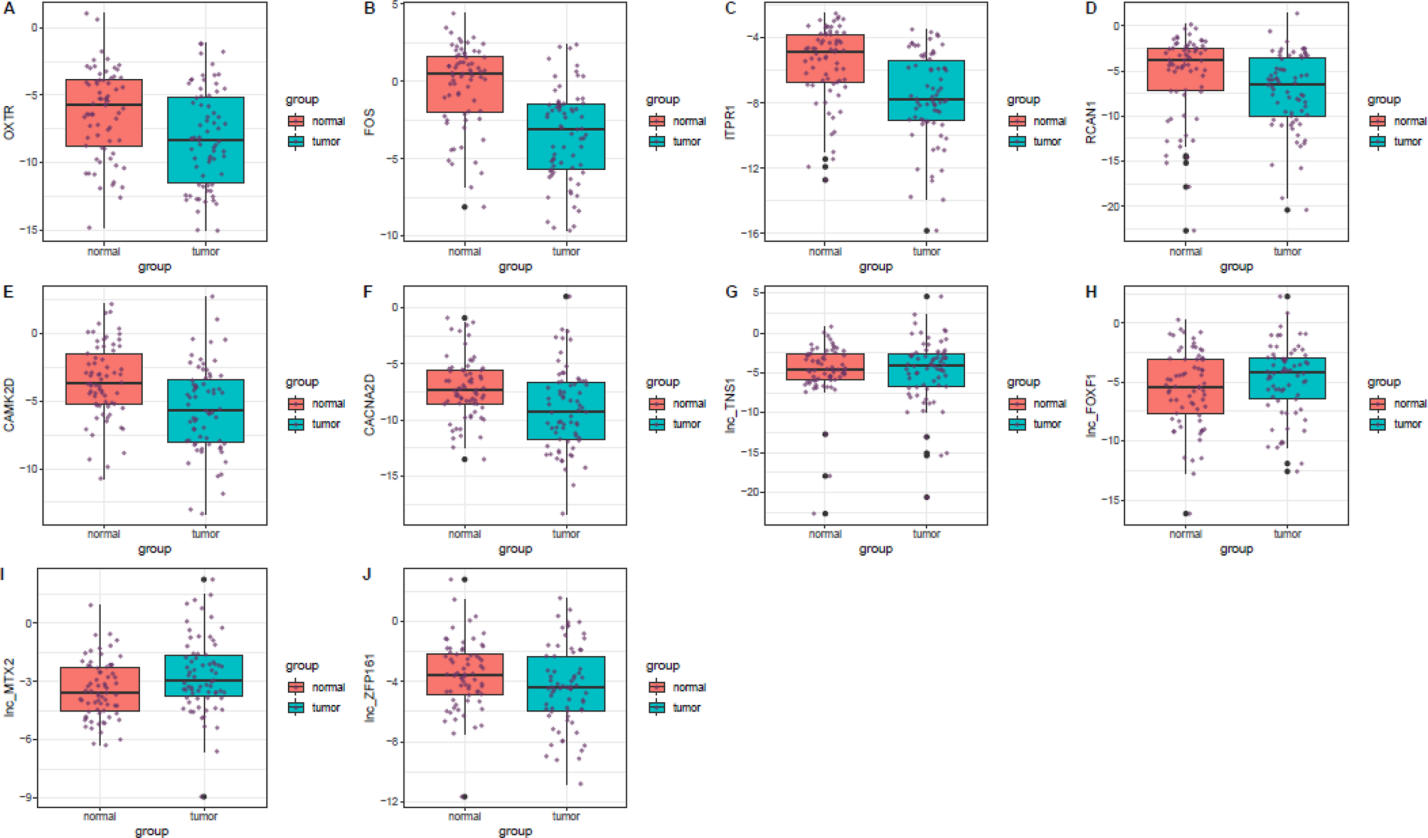
Relative expression levels of oxytocin related genes in breast cancer samples and nearby non-cancerous tissues. Minimum, maximum and interquartile range are shown. Purple dots represent expression of each gene in each sample. Outliers are shown as black dots.

Figure 3 depicts the relative expression levels of oxytocin related genes in breast cancer samples and nearby non-cancerous tissues.

Expression levels of *OXTR*, *FOS*, *ITPR1*, *RCAN1*, *CAMK2D*, *CACNA2D* and *lnc_ZFP161* were significantly down-regulated in the breast cancer tissues compared with nearby non-cancerous tissues. On the other hand, expression of *lnc_MTX2* was higher in breast cancer tissues compared with controls. Expressions of *lnc_TNS1* and *lnc_FOXF1* were not different between these two kinds of samples (Table 2).

**Table 2:** Detailed parameters of expression analysis of oxytocin-related genes in breast cancer samples compared with nearby non-cancerous tissues.

Genes	SE	Ration of mean expression	P-value	95% CI	
*OXTR*	0.51	0.30	**9.70E−04**	− 2.76	− 0.74
*FOS*	0.48	0.11	**7.95E−09**	− 4.13	− 2.21
*ITPR1*	0.39	0.24	**1.40E−06**	− 2.86	− 1.29
*RCAN1*	0.42	0.30	**8.50E−05**	− 2.59	− 0.92
*CAMK2D*	0.37	0.25	**6.28E−07**	− 2.74	− 1.28
*CACNA2D*	0.44	0.27	**5.56E−05**	− 2.77	− 1.01
*lnc_TNS1*	0.47	1.08	8.16E**−**01	− 0.83	1.05
*lnc_FOXF1*	0.44	1.69	8.81E**−**02	− 0.12	1.63
*lnc_MTX2*	0.24	1.77	**1.08E−03**	0.34	1.30
*lnc_ZFP161*	0.31	0.58	**1.39E−02**	− 1.41	− 0.17

### Association between expression of genes and clinical data

Then, we appraised the association between expression levels of oxytocin-associated genes and a number of clinical and demographic data such as cancer stage and grade, age, mitotic rate, tumor size and hormone receptor status. Expression of *CACNA2D* was associated with mitotic rate and PR status (P values = 3.02E−02 and 2.53E−02, respectively). Expression of other oxytocin-related genes was not associated with these parameters (Table 3).

**Table 3:** Association between expression of oxytocin-related genes and clinical/demographic data.

		*OXTR*				*FOS*				*ITPR1*				*RCAN1*				*CAMK2D*			
		Down regulated	Same	Up regulated	P-value	Downregulated	Same	Up regulated	P-value	Down regulated	Same	Up regulated	P-value	Down regulated	Same	Up regulated	P-value	Down regulated	Same	Up regulated	P-value
**Age**					2.99E−01				5.30E−01				1.69E−01				6.18E−01				7.16E−01
	Post-menopause	0.56	0.06	0.22		0.69	0.09	0.06		0.53	0.22	0.09		0.09	0.03	0.03		0.57	0.27	0.03	
	Pre-menopause	0.06	0.00	0.09		0.13	0.00	0.03		0.09	0.00	0.06		0.66	0.13	0.06		0.07	0.07	0.00	
Stage					5.83E−01				4.33E−01				6.18E−01				5.86E−01				3.39E−01
	0	0.03	0.00	0.02		0.05	0.00	0.00		0.02	0.03	0.00		0.02	0.02	0.02		0.00	0.03	0.02	
	1	0.19	0.03	0.06		0.17	0.05	0.06		0.19	0.06	0.03		0.20	0.05	0.03		0.16	0.10	0.02	
	2	0.22	0.03	0.05		0.27	0.03	0.00		0.20	0.05	0.05		0.19	0.08	0.03		0.16	0.08	0.06	
	3	0.13	0.05	0.14		0.23	0.03	0.05		0.17	0.08	0.06		0.19	0.03	0.09		0.18	0.10	0.03	
	4	0.05	0.00	0.02		0.06	0.00	0.00		0.03	0.03	0.00		0.05	0.02	0.00		0.06	0.00	0.00	
Histological grade					6.41E−01				6.61E−01				3.89E−01				9.44E−01				6.08E−01
	0	0.00	0.00	0.02		0.02	0.00	0.00		0.02	0.00	0.00		0.02	0.00	0.00		0.00	0.02	0.00	
	1	0.13	0.02	0.05		0.13	0.05	0.02		0.10	0.05	0.05		0.12	0.05	0.03		0.12	0.05	0.02	
	2	0.27	0.07	0.18		0.38	0.05	0.08		0.35	0.08	0.08		0.32	0.08	0.12		0.29	0.14	0.10	
	3	0.18	0.03	0.05		0.23	0.02	0.02		0.13	0.12	0.02		0.18	0.05	0.03		0.14	0.10	0.02	
Mitotic rate					3.19E−01				7.77E−01				6.03E−01				6.87E−01				5.14E−01
	0	0.00	0.00	0.02		0.02	0.00	0.00		0.02	0.00	0.00		0.02	0.00	0.00		0.00	0.02	0.00	
	1	0.24	0.04	0.15		0.31	0.04	0.07		0.30	0.06	0.07		0.26	0.11	0.06		0.27	0.13	0.04	
	2	0.31	0.06	0.06		0.35	0.06	0.02		0.24	0.11	0.07		0.28	0.04	0.11		0.25	0.10	0.08	
	3	0.07	0.00	0.06		0.11	0.00	0.02		0.07	0.06	0.00		0.09	0.02	0.02		0.06	0.06	0.00	
Tumor size					3.96E−01				5.55E−01				6.32E−01				8.48E−01				7.74E−01
	< 2	0.18	0.07	0.05		0.20	0.07	0.03		0.20	0.08	0.02		0.22	0.05	0.03		0.17	0.10	0.02	
	2–5	0.42	0.05	0.22		0.55	0.05	0.08		0.40	0.15	0.13		0.42	0.13	0.13		0.40	0.19	0.10	
	> 5	0.02	0.00	0.00		0.02	0.00	0.00		0.02	0.00	0.00		0.02	0.00	0.00		0.02	0.00	0.00	
ER status					4.46E−01				9.07E−01				7.52E−01				9.51E−01				1.87E−01
	Positive	0.50	0.07	0.24		0.15	0.02	0.02		0.11	0.04	0.04		0.11	0.04	0.04		0.06	0.10	0.04	
	Negative	0.15	0.00	0.04		0.65	0.11	0.06		0.52	0.20	0.09		0.52	0.17	0.13		0.50	0.21	0.10	
PR status					6.99E−02				9.89E−01				8.94E−01				3.74E−01				5.35E−01
	Positive	0.15	0.06	0.06		0.21	0.04	0.02		0.15	0.08	0.04		0.15	0.04	0.08		0.16	0.06	0.06	
	Negative	0.49	0.02	0.23		0.58	0.09	0.06		0.47	0.17	0.09		0.47	0.17	0.09		0.41	0.24	0.08	
Her2 status					1.83E−01				1.86E−01				8.23E−01				8.57E−02				6.67E−01
	Positive	0.20	0.06	0.13		0.35	0.04	0.00		0.26	0.09	0.04		0.24	0.04	0.11		0.25	0.10	0.06	
	Negative	0.44	0.02	0.15		0.44	0.09	0.07		0.37	0.15	0.09		0.39	0.17	0.06		0.31	0.21	0.08	
Menarche age					5.52E−01				8.05E−01				5.09E−01				2.24E−01				9.68E−02
	10–12	0.23	0.02	0.05		0.25	0.03	0.02		0.20	0.03	0.07		0.22	0.07	0.02		0.14	0.16	0.02	
	13–15	0.35	0.07	0.20		0.47	0.07	0.08		0.38	0.15	0.08		0.40	0.07	0.15		0.38	0.10	0.12	
	16–18	0.05	0.00	0.03		0.07	0.00	0.02		0.05	0.03	0.00		0.03	0.03	0.02		0.05	0.03	0.00	
Menopause age					6.11E−01				5.85E−01				1.44E−01				9.07E−01				4.24E−01
	< = 50	0.44	0.03	0.19		0.56	0.06	0.03		0.38	0.22	0.06		0.47	0.13	0.06		0.43	0.17	0.03	
	51–55	0.13	0.03	0.13		0.19	0.03	0.06		0.19	0.00	0.09		0.22	0.03	0.03		0.13	0.17	0.00	
	> = 56	0.06	0.00	0.00		0.06	0.00	0.00		0.06	0.00	0.00		0.06	0.00	0.00		0.07	0.00	0.00	
Breast feeding duration					3.08E−01				2.62E−01				2.21E−01				1.81E−01				6.44E−01
	0	0.07	0.03	0.10		0.10	0.05	0.05		0.08	0.05	0.07		0.12	0.02	0.07		0.10	0.07	0.03	
	1–30	0.20	0.05	0.05		0.22	0.05	0.03		0.18	0.07	0.05		0.20	0.05	0.05		0.17	0.09	0.03	
	31–60	0.17	0.03	0.05		0.22	0.00	0.03		0.12	0.10	0.03		0.12	0.12	0.02		0.10	0.12	0.03	
	61–120	0.15	0.00	0.03		0.17	0.02	0.00		0.15	0.03	0.00		0.13	0.02	0.03		0.10	0.03	0.03	
	> = 121	0.03	0.00	0.03		0.07	0.00	0.00		0.07	0.00	0.00		0.05	0.00	0.02		0.07	0.00	0.00	
Hormone replacement therapy					5.43E−01				6.75E−01				1.18E−01				6.02E−01				7.24E−01
	Yes	0.13	0.00	0.05		0.13	0.04	0.02		0.15	0.00	0.04		0.13	0.02	0.04		0.11	0.04	0.02	
	No	0.51	0.09	0.22		0.67	0.09	0.05		0.47	0.25	0.09		0.49	0.20	0.13		0.43	0.28	0.11	

		*CACNA2D*				*lnc_TNS1*				*lnc_FOXF1*				*lnc_MTX2*				*lnc_ZFP161*			
		Down regulated	Same	Up regulated	P-value	Down regulated	Same	Up regulated	P-value	Down regulated	Same	Up regulated	P-value	Down regulated	Same	Up regulated	P-value	Down regulated	Same	Up regulated	P-value
**Age**					2.54E−01				3.18E−01				5.03E−01				5.76E−01				9.06E−01
	Post-menopause	0.06	0.03	0.06		0.31	0.25	0.28		0.25	0.19	0.41		0.03	0.03	0.09		0.09	0.03	0.03	
	Pre-menopause	0.56	0.19	0.09		0.06	0.00	0.09		0.06	0.00	0.09		0.09	0.38	0.38		0.44	0.25	0.16	
Stage					3.17E−01				3.58E−01				9.40E−01				1.43E−01				4.60E−01
	0	0.02	0.02	0.02		0.00	0.02	0.03		0.02	0.00	0.03		0.02	0.00	0.03		0.02	0.02	0.02	
	1	0.20	0.06	0.02		0.08	0.11	0.09		0.06	0.06	0.16		0.05	0.16	0.08		0.11	0.09	0.08	
	2	0.19	0.08	0.03		0.16	0.06	0.08		0.11	0.05	0.14		0.06	0.09	0.14		0.19	0.05	0.06	
	3	0.11	0.11	0.09		0.09	0.08	0.14		0.11	0.05	0.16		0.06	0.05	0.20		0.13	0.11	0.08	
	4	0.05	0.00	0.02		0.05	0.00	0.02		0.03	0.02	0.02		0.00	0.05	0.02		0.06	0.00	0.00	
Histological grade					5.71E−01				6.24E−01				6.21E−01				5.79E−01				4.15E−01
	0	0.00	0.02	0.00		0.00	0.00	0.02		0.00	0.00	0.02		0.00	0.00	0.02		0.00	0.00	0.02	
	1	0.13	0.03	0.03		0.07	0.08	0.05		0.10	0.02	0.08		0.07	0.05	0.08		0.13	0.05	0.02	
	2	0.27	0.17	0.08		0.20	0.13	0.18		0.15	0.10	0.27		0.08	0.18	0.25		0.22	0.17	0.13	
	3	0.15	0.05	0.07		0.08	0.05	0.13		0.05	0.05	0.17		0.02	0.10	0.15		0.12	0.07	0.08	
Mitotic rate					**3.02E−02**				5.67E−01				5.53E−01				3.53E−01				4.32E−01
	0	0.00	0.02	0.00		0.00	0.00	0.02		0.00	0.00	0.02		0.00	0.00	0.02		0.00	0.00	0.02	
	1	0.24	0.17	0.02		0.19	0.07	0.17		0.17	0.02	0.24		0.07	0.09	0.26		0.24	0.13	0.06	
	2	0.30	0.04	0.09		0.15	0.15	0.13		0.13	0.07	0.22		0.07	0.22	0.13		0.17	0.11	0.15	
	3	0.06	0.02	0.06		0.04	0.02	0.07		0.02	0.04	0.07		0.02	0.04	0.07		0.06	0.04	0.04	
Tumor size					4.34E−01				1.29E−01				6.80E−01				2.66E−01				2.62E−01
	< 2	0.22	0.07	0.02		0.07	0.13	0.10		0.08	0.05	0.17		0.07	0.12	0.12		0.10	0.13	0.07	
	2–5	0.35	0.18	0.15		0.32	0.12	0.25		0.23	0.12	0.33		0.10	0.25	0.33		0.38	0.13	0.17	
	> 5	0.02	0.00	0.00		0.02	0.00	0.00		0.02	0.00	0.00		0.02	0.00	0.00		0.02	0.00	0.00	
ER status					9.23E−01				1.47E−01				2.42E−01				6.79E−01				1.58E−01
	Positive	0.09	0.06	0.04		0.06	0.02	0.11		0.26	0.17	0.39		0.04	0.04	0.11		0.06	0.06	0.07	
	Negative	0.46	0.20	0.15		0.30	0.28	0.24		0.06	0.00	0.13		0.15	0.28	0.39		0.48	0.20	0.13	
PR status					**2.53E−02**				8.69E−02				4.02E−01				6.09E−01				5.55E−01
	Positive	0.11	0.04	0.11		0.08	0.04	0.15		0.08	0.02	0.17		0.06	0.06	0.15		0.11	0.08	0.08	
	Negative	0.43	0.23	0.08		0.28	0.26	0.19		0.25	0.15	0.34		0.13	0.26	0.34		0.43	0.17	0.13	
Her2 status					5.59E−01				3.76E−01				2.75E−01				9.60E−01				2.97E−01
	Positive	0.22	0.07	0.09		0.17	0.07	0.15		0.17	0.04	0.19		0.07	0.13	0.19		0.24	0.06	0.09	
	Negative	0.33	0.19	0.09		0.19	0.22	0.20		0.15	0.13	0.33		0.11	0.19	0.31		0.30	0.20	0.11	
Menarche age					5.22E−02				2.85E−01				2.59E−01				7.89E−01				7.09E−01
	10–12	0.17	0.12	0.02		0.12	0.10	0.08		0.13	0.08	0.08		0.07	0.12	0.12		0.17	0.07	0.07	
	13–15	0.38	0.07	0.17		0.23	0.17	0.22		0.17	0.08	0.37		0.12	0.20	0.30		0.32	0.17	0.13	
	16–18	0.05	0.03	0.00		0.02	0.00	0.07		0.02	0.02	0.05		0.00	0.03	0.05		0.02	0.03	0.03	
Menopause age					3.25E−01				6.68E−01				8.60E−01				3.01E−01				9.24E−01
	< = 50	0.44	0.09	0.13		0.22	0.22	0.22		0.19	0.16	0.31		0.06	0.34	0.25		0.34	0.19	0.13	
	51–55	0.13	0.13	0.03		0.13	0.03	0.13		0.09	0.03	0.16		0.06	0.03	0.19		0.16	0.06	0.06	
	> = 56	0.06	0.00	0.00		0.03	0.00	0.03		0.03	0.00	0.03		0.00	0.03	0.03		0.03	0.03	0.00	
Breast feeding duration					1.28E−01				7.97E−01				7.26E−01				4.57E−01				9.61E−01
	0	0.10	0.05	0.05		0.07	0.05	0.08		0.05	0.07	0.08		0.07	0.03	0.10		0.12	0.03	0.05	
	1–30	0.20	0.02	0.08		0.13	0.07	0.10		0.12	0.07	0.12		0.05	0.15	0.10		0.15	0.08	0.07	
	31–60	0.12	0.13	0.00		0.07	0.08	0.10		0.08	0.02	0.15		0.03	0.12	0.10		0.12	0.08	0.05	
	61–120	0.12	0.03	0.03		0.07	0.07	0.05		0.05	0.03	0.10		0.03	0.05	0.10		0.08	0.05	0.05	
	> = 121	0.03	0.02	0.02		0.05	0.00	0.02		0.03	0.00	0.03		0.02	0.00	0.05		0.05	0.02	0.00	
Hormone replacement therapy					3.88E−01				6.07E−01				2.34E−01				6.30E−01				4.69E−01
	Yes	0.13	0.02	0.04		0.05	0.04	0.09		0.05	0.00	0.13		0.04	0.04	0.11		0.13	0.02	0.04	
	No	0.42	0.25	0.15		0.31	0.24	0.27		0.27	0.16	0.38		0.15	0.29	0.38		0.42	0.22	0.18	

### ROC curves

**Figure 4:**
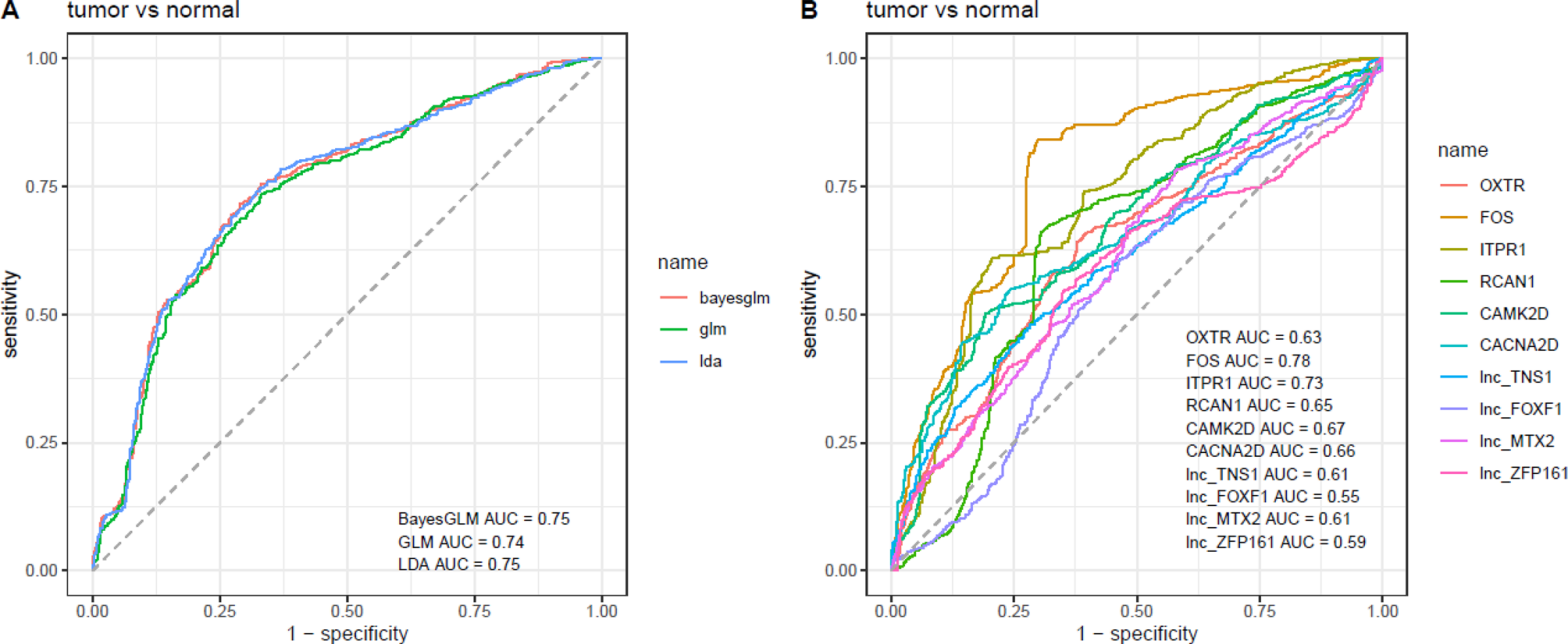
Efficacy of three predictive models in predicting the diagnostic power of oxytocin-related genes **(A)** and the obtained AUC values for each gene **(B)**.

Figure 4 demonstrates the efficacy of three predictive models in predicting the diagnostic power of oxytocin-related genes and the obtained AUC values for each gene. ROC curves were depicted using Log 2 values of transcript quantities of all genes as the predictive features to train three machine learning models (LDA, BayesGLM and GLM) with tenfold cross validation. AUC metric was computed to pick the best model. Finally, BayesGLM model was selected based on the previous test, and the model was trained for each gene separately to test the distinguishing power of specific genes.

*FOS* and *ITPR1* had the highest AUC value among the oxytocin-related genes. Combination of expression profile of all oxytocin-related genes increased the AUC value to 0.75. However, the combinatorial sensitivity and specificity values were lower than some individual genes (Table 4).

**Table 4:** Detailed characteristics of ROC curves.

Genes	AUC	Sensitivity	Specificity
*OXTR*	0.63	0.64	0.62
*FOS*	0.78	0.84	0.70
*ITPR1*	0.73	0.61	0.80
*RCAN1*	0.65	0.66	0.70
*CAMK2D*	0.67	0.50	0.81
*CACNA2D*	0.66	0.55	0.76
*lnc_TNS1*	0.61	0.48	0.72
*lnc_FOXF1*	0.55	0.61	0.54
*lnc_MTX2*	0.61	0.78	0.42
*lnc_ZFP161*	0.59	0.55	0.65
All genes	0.75	0.72	0.71

**Figure 5:**
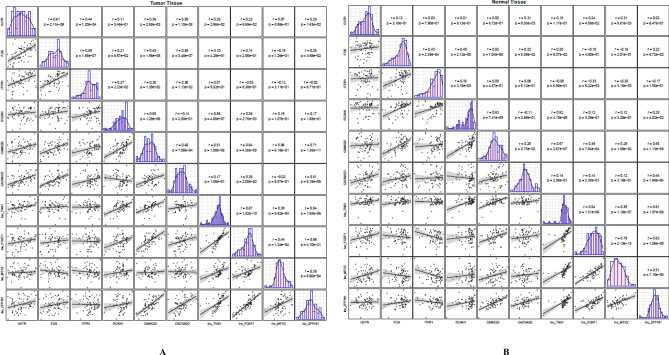
Correlations between expression levels of oxytocin-associated genes.

In breast cancer tissues, the most robust correlations have been detected between *lnc_ZFP161*/ *lnc_FOXF1*, *CAMK2D*/ *lnc_ZFP161* and *CAMK2D* / *lnc_FOXF1* (r = 0.86, 0.71 and 0.64 respectively) (Fig. 5A). In the non-cancerous tissues, the strongest correlation was detected between *lnc_FOXF1/lnc_MTX2 and lnc_ZFP161*/*CAMK2D* respectively (r = 0.78 and 0.65). (Fig. 5B).

## Discussion

Breast cancer is a complex disorder in which several molecular mechanisms are involved. Immunology regulations may also affect breast cancer development and immunodeficiency may promote adaptive alterations of host gut- and tissue-based microbiome^12^. LncRNAs can affect several aspects in this regard. Several lines of evidence such as the structural and genomic relation to vasopressin, co-expression of oxytocin and vasopressin, and the mitogenic effects of these hormones connected oxytocin to carcinogenesis^1^. Moreover, breastfeeding has been shown to decrease the risk of a number of cancers and particularly breast cancer, with elongated periods of breastfeeding being associated with a progressive reduction in the risk of this cancer^13, 14^. Meanwhile, oxytocin has been shown to affect immune regulation^15^, thus possibly influencing breast cancer pathogenesis via different routes. Despite these observations, the underlying mechanisms involving oxytocin in breast carcinogenesis are not elucidated. Based on the importance of lncRNAs in the regulation of cancer-related pathways, we aimed at identifying the oxytocin-related lncRNAs through an in silico approach and assessed expression of a number of oxytocin-related mRNAs and lncRNAs in breast cancer samples. We identified down-regulation of *OXTR*, *FOS*, *ITPR1*, *RCAN1*, *CAMK2D*, *CACNA2D* and *lnc_ZFP161*, and up-regulation of *lnc_MTX2* in the breast cancer tissues compared with nearby non-cancerous tissues. In line with our observation, Ariana et al. have reported lower expression of OXTR in breast cancer tissues compared with para-cancerous tissues. They also detected high expression of oxytocin in breast cancer patients^16^. Based on the results of in vitro and in vivo investigations regarding the role of oxytocin as a peptide with bivariate biological functions, Imanieh et al. have hypothesized that oxytocin acts as either an activator or inhibitor of growth through activating OXTR in tumoral cells^17^. The observed down-regulation of *FOS* in breast cancer samples is in line with the study of Fisler, which reported association between higher FOS expression and better survival of patients with breast cancer. Moreover, higher levels of FOS target apoptosis-effector gene have been associated with improved survival of these patients. Based on these results, authors have suggested that FOS is a pro-apoptotic protein^18^. In addition to the functional association with oxytocin-related pathways, *ITPR1* has a regulatory role on autophagy and sensitivity to chemotherapeutic agents in cancer cells^19^. Therefore, its down-regulation in breast cancer cells might influence several aspects of breast carcinogenesis. RCAN1 has been suggested as a super-enhancer-driven tumor suppressor whose down-regulation enhances the malignant features of breast cancer cells^20^. CAMK2D is a kinase that regulates several cellular processes, such as proliferation, differentiation and apoptosis. Chi et al. have reported higher levels of CAMK2D expression and phosphorylation in breast cancer samples compared with non-cancerous samples^21^. This finding is in contrast with the reported expression pattern of *CAMK2D* mRNA in the current study. Further assessment of expression levels of this gene at both mRNA and protein levels is necessary for solving this controversy. We also detected down-regulation of the calcium channel coding gene *CACNA2D* in breast cancer samples and its association with mitotic rate and PR status. Former studies have reported that breast cancer cells can attain a selective growth advantage through modulating ion channel expression or function. These channels have also been shown to participate in the prominent features of this cancer^22^. However, the specific role of CACNA2D has not been elucidated. Future functional studies are required to clarify this point.

We also assessed the diagnostic value of oxytocin-related genes in breast cancer. *FOS* and *ITPR1* had the highest AUC value among the oxytocin-related genes. Combination of expression profile of all oxytocin-related genes increased the AUC value to 0.75. However, the combinatorial sensitivity and specificity values were lower than some individual genes. We recommend appraisal of expression of these genes in the peripheral blood of patients with breast cancer to unravel their diagnostic potential.

Finally, appraisal of correlation between expression levels of oxytocin-related genes has led to identification of specific patterns in cancerous and non-cancerous tissues. In breast cancer tissues, the most robust correlations have been detected between *lnc_ZFP161*/*lnc_FOXF1*, *CAMK2D*/*lnc_ZFP161* and *CAMK2D*/*lnc_FOXF1*. In the non-cancerous tissues, the strongest correlation was detected between *lnc_FOXF1/Lnc_MTX2* followed by *lnc_ZFP161* and *CAMK2D*. Taken together, oxytocin-associated genes have been dysregulated in breast cancer tissues. Moreover, the correlation between these genes is influenced by the presence of cancer, as correlation coefficients between gene pairs were different in tumoral and non-tumoral tissues.

The current study used a combination of bioinformatics and gene expression methods. Bioinformatics methods have been extensively used to find appropriate targets for experimental assessment of gene expressions. A common strategy is to collect all related public expression-profiling of microarray and RNA-sequencing data using appropriate criteria and to combine them to construct co-expression network to identify hub mRNA/lncRNAs along with using PPI network analysis^23, 24^. However, in the current study, we only selected one dataset. Although selection of this dataset was based on the appropriateness of included samples and methods, additional datasets could also be used for this purpose. So, we proposed future assessment of the results of this study using these datasets.

Although deep learning method is a very promising way to predict prognosis for cancer based on biomarkers, an important prerequisite for efficient deep learning models is the large number of samples in proportion of the number of parameters in the model. Here, in the statistical part of the study, we aimed to validate the selected markers in a case–control study with 69 specimens. So, some simpler machine learning methods were used to examine the efficacy of markers. Finally, the potential causal effects behind the association of the oxytocin-related lncRNA biomarkers with breast cancer should be verified using a statistical approach named Mendelian Randomization^25^.

Taken together, our study demonstrates abnormal expression levels of oxytocin-related genes in breast cancer tissues versus non-cancerous tissues and influence of cancer on the correlation network between these genes, potentiating these genes as biomarkers for breast cancer.
